# Beyond Rational Decision-Making: Modelling the Influence of Cognitive Biases on the Dynamics of Vaccination Coverage

**DOI:** 10.1371/journal.pone.0142990

**Published:** 2015-11-23

**Authors:** Marina Voinson, Sylvain Billiard, Alexandra Alvergne

**Affiliations:** 1 Université de Lille—Sciences et Technologies, UMR 8198 Evo-Eco-Paleo, Villeneuve d'Ascq, France; 2 School of Anthropology and Museum Ethnography, University of Oxford, Oxford, United Kingdom; University of Waterloo, CANADA

## Abstract

**Background:**

Theoretical studies predict that it is not possible to eradicate a disease under voluntary vaccination because of the emergence of non-vaccinating “free-riders” when vaccination coverage increases. A central tenet of this approach is that human behaviour follows an economic model of rational choice. Yet, empirical studies reveal that vaccination decisions do not necessarily maximize individual self-interest. Here we investigate the dynamics of vaccination coverage using an approach that dispenses with payoff maximization and assumes that risk perception results from the interaction between epidemiology and cognitive biases.

**Methods:**

We consider a behaviour-incidence model in which individuals perceive actual epidemiological risks as a function of their opinion of vaccination. As a result of confirmation bias, sceptical individuals (negative opinion) overestimate infection cost while pro-vaccines individuals (positive opinion) overestimate vaccination cost. We considered a feedback between individuals and their environment as individuals could change their opinion, and thus the way they perceive risks, as a function of both the epidemiology and the most common opinion in the population.

**Results:**

For all parameter values investigated, the infection is never eradicated under voluntary vaccination. For moderately contagious diseases, oscillations in vaccination coverage emerge because individuals process epidemiological information differently depending on their opinion. Conformism does not generate oscillations but slows down the cultural response to epidemiological change.

**Conclusion:**

Failure to eradicate vaccine preventable disease emerges from the model because of cognitive biases that maintain heterogeneity in how people perceive risks. Thus, assumptions of economic rationality and payoff maximization are not mandatory for predicting commonly observed dynamics of vaccination coverage. This model shows that alternative notions of rationality, such as that of ecological rationality whereby individuals use simple cognitive heuristics, offer promising new avenues for modelling vaccination behaviour.

## Introduction

Vaccination has greatly reduced the burden of infectious diseases worldwide. However, by 2015, only smallpox has been eradicated by programs of voluntary vaccination and global outbreaks of measles, mumps, whooping cough, polio and rubella are repeatedly being reported in both developed and developing regions. Why voluntary vaccination programs succeed or fail is contingent on the acceptance or rejection of vaccines by individuals; considering the vaccination decision-making process is thus pivotal for making inferences about the dynamics of vaccination coverage and disease transmission. Over the last decade, a number of theoretical studies explicitly modelled human behaviour as a key parameter for determining how vaccines scares unfold (reviewed in [1,2]). This led to the new field of behavioural epidemiology [3] and the development of “prevalence-based” models [1] positing that individuals adjust their decisions based on an information relating to the number of infected individuals. Assuming that individuals are rational, that is, agents that optimize their utility i.e. individuals make a logical and coherent choice depending on choices of others, it was concluded that it is not possible to eradicate a disease under voluntary vaccination because of the emergence of non-vaccinating free riders when vaccination coverage is sufficiently high [4–6]. This approach is at odds with psychological and anthropological literature on human decision-making and insufficient if we are to explain why some vaccine preventable diseases can be either globally eradicated (e.g. small pox) or locally eliminated (e.g. polio in India), or why individuals refuse vaccination despite scientific evidence that it is safe (reviewed in [7]). Here we propose an alternative to classic behavioural epidemiology studies by considering a revised version of rationality, that of an ecological rationality [8,9], enabled by the mind’s “adaptive toolbox” [10]. This framework dispenses with optimization and with complex calculations of utilities altogether and views decision-making as the expression of evolved cognitive dispositions (e.g. heuristics or rules of thumb including social learning abilities). This enables us to develop a behaviour-incidence model explicitly considering cognitive mechanisms with the aim to investigate how the feedback between cognition and epidemiology may influence the dynamic of disease transmission.

How do people decide whether or not to vaccinate? A key factor is the perception of risks associated with infection and vaccination [11]. Risk perception is central to most health behaviour theories and in this line, a recent meta-analysis [12] based on > 15,000 individuals revealed a significant association (measured by a summary effect size *r*, which was obtained from pooling effect sizes across studies) between vaccine uptake and (i) the perceived likelihood of infection (12 studies, *r* (summary effect size [range]) = 0.26 [-0.12; 0.45]), (ii) the susceptibility to illness (5 studies, *r* = 0.24 [0.15; 0.36]) and to a lesser extent, (iii) the severity of the disease (31 studies, *r* = 0.16 [-0.18; 0.39]) (meta-analysis [12]). In classic behavioural epidemiology, risk perception relates to the prevalence of infected individuals at a given time, either in the general population (non-structured population and homogeneously mixed population) or in a social network [13,14]. This underlying game theory framework considers that individuals make decisions based on an objective evaluation of the epidemiology, computing statistics using a large data size (but see [15–17]). However, this assumption is problematic for at least two reasons. First, human cognition has been demonstrated to be inefficient at dealing with uncertainty and computing probability [10]; rather, individuals may use empirical knowledge based on a small number of cases. Second, risk assessment is likely to be partly independent from infection [1] as it is shaped by a number of psychological and social factors that may lead to an under- or an over-estimation of epidemiological risks, including media coverage [18], salient previous experience [11], belief about contagion [19], family and peers [11,20] and trust in providers, medical professionals and the state [7,21]. To evaluate temporal and spatial variation in vaccine uptake, one must thus refine assumptions of rationality to consider, not only change in epidemiology, but also how the evolved cognition responds to features of the social and epidemiological environments.

An alternative to current modelling of vaccination decision-making may come from the consideration of evolutionary-ecological models, focusing on the concept of ecological rationality [9,22]. In this framework, cognitive dispositions are evolved, i.e. decision rules have been selected for, but the resulting behaviour may or may not be adaptive, i.e. maximizing current individual survival and reproductive success. It follows that to understand the processes underlying vaccination decision-making, one focuses on the environment and the cognitive mechanisms underlying decisions rather than health outcomes of decisions. Proponents of ecological rationality argue that humans have evolved heuristics, i.e. simple cognitive rules processing a few pieces of information available from the environment [9]. For the purpose of this paper, which aims to investigate the role of cognitive biases for predicting commonly observed dynamics of vaccination coverage such as the failure to reach herd immunity and oscillations between high and low levels of coverage, we focus on those heuristics or cognitive shortcuts that are likely to allow for heterogeneity and change in opinion about vaccination. In modelling how individuals interpret epidemiological information, we first decided to consider “confirmation bias”, i.e. the propensity to look for evidence supporting pre-existing beliefs while considering disconfirmatory evidence with great scrutiny [23,24]. Confirmation bias has been observed in the context of pertussis vaccination for children: when presented with the same risk-benefit information about vaccination, non-vaccinators became less committed with vaccination, while vaccinators became more committed [25]. The second heuristic we considered is “imitate the majority” or conformism, as the role of peer influence has repeatedly been invoked for understanding vaccination behaviour [7,26,27].

In this paper, we consider a behaviour-incidence model in which opinion formation mediates a feedback between vaccination behaviour and the disease. As compared to previous behaviour-incidence models, our approach does not make use of game theory analysis (see also [28,29]). We first consider that disease incidence and vaccination coverage determine how many people suffer from negative effects, an information that represents epidemiological costs. We then assume that an individual interprets those costs as a function of their opinion of vaccination. Specifically, a sceptical individual will be more likely to overestimate a vaccination cost while a pro-vaccine advocate will more likely to overestimate the cost of infection. It follows that given the same epidemiological information, individuals will vaccinate at different rates depending on their a priori opinion. Finally, we consider that individuals respond to their environment over time as they can change their opinion as a function of both epidemiological costs and the most common opinion in the population (conformism). The model assumes that the population mixes homogenously so that anyone can infect any other individual in the population and the information on epidemiological costs is globally available to everyone through the media. The goal of this study is to investigate how the interaction between human cognition and both the epidemiological and social environments shape the dynamics of vaccination coverage.

## Methods

### Rationale

**Fig 1:**
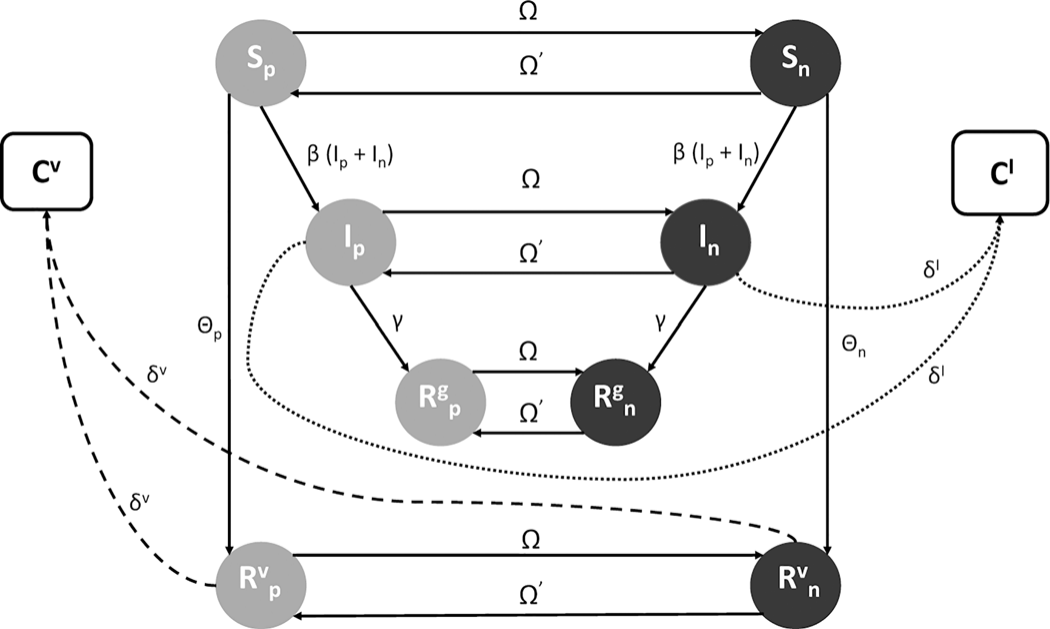
Structure of the behaviour-incidence model. The model is an augmentation of a classic SIR compartmental model [30]. Individuals are characterized by both their epidemiological status (*S*: susceptible; *I*: infected; *R* ^*v*^: recovered through vaccination; *R* ^*g*^: recovered naturally) and for each compartment, their opinion of vaccination (positive: subscript p; non-shaded colours; negative: subscript n; shaded colours). *C* ^*V*^ and *C* ^*I*^ are compartments that indicate the total recalled number of individuals having suffered negative side effects from vaccination and infection, respectively. *β* indicates the rate of infection transmission; Ω indicates the rate at which individuals change their opinion (from positive to negative Ω or from negative to positive Ω′); *δ* indicates the number of individuals suffering side effects from either vaccination *δ* ^*V*^ and infection *δ* ^*I*^; *θ* indicates the rate at which individuals vaccinate. Each individual dies at the same rate *d*.

The model corresponds to a SIR (susceptible-infected-recovered/immune) compartmental model capturing the disease transmission process [30] augmented with a belief model (Fig 1). It enables individuals to be characterized by both their epidemiological status and their opinion. Specifically, individuals can belong to one of 4 epidemiological compartments: susceptible, infected, immune through vaccination and immune naturally. In each compartment, individuals can be characterized either as “pro-vaccines” (positive opinion) or “sceptical” (negative opinion). Across time, individuals can change compartments: for instance, susceptible individuals may become infected, or they may choose to vaccinate. The system of compartments and flows between them is defined by a system of ordinary differential equations (see section 2.iii) to examine numerical simulations at different values of the parameters.

The rate at which individuals become vaccinated is a function of both the epidemiological costs associated with infection and vaccination and the distribution of opinions of vaccination in the population. First, costs are defined by the epidemiology with the number of infected individuals and vaccination coverage determining the number of people suffering negative side effects from infection and vaccination, respectively. Individuals are assumed to vaccinate more quickly when the infection cost is high and more slowly when the vaccination cost is high. Then, given the same epidemiological information on costs, individuals with different opinions vaccinate at different rates. We considered that pro-vaccines individuals give more weight to the role of infection cost in their decision process, while sceptics give more weight to the vaccination cost (due to confirmation bias). Thus, while pro-vaccines individuals always take up vaccines more quickly than sceptics, the difference between them in how rapidly they take up vaccination is considered to be the highest for levels of vaccination coverage that are either low (high infection cost) or high (high vaccination cost). Finally, individuals can change their opinion as a function of both the epidemiology and the most common opinion in the population.

### The full model

#### The structure (Fig 1)

We considered the case of a single contagious disease, which does not increase the death rate, but which causes negative side effects for infected individuals. Our goal here is not to study a disease in particular but to provide general insights with regard to the effect of opinion on the dynamics of infection and vaccination coverage. We used a compartmental epidemiological model with births and deaths [30] in which the population is divided into 4 compartments: the number of susceptible (*S*), the number of infected (*I*), the number of immune through vaccination (*R* ^*v*^) and the number of immune through natural immunization (*R* ^*g*^). A susceptible individual becomes infected at the rate *β* (the transmission rate) and vaccinates (transits from compartment *S* to *R* ^*v*^) at the rate *θ*. Individuals give births to susceptible individuals at the rate *b* and each individual can die at the rate *d = b*, so that the population remains of constant size. Then, for each compartment, the population is divided into 2 subgroups, with individuals having either a positive opinion of vaccination (pro-vaccines individuals, subscript p) or a negative opinion (sceptical individuals, subscript n). The formation of the opinion and the decision to vaccinate are two separated processes that both depend on the environment. Individuals can change their opinion during their lifetime, and given their opinion at a given time, they decide to get vaccinated or not. At any given time, the total recalled number of individuals having suffered negative side effects from becoming infected or vaccinated constitute a globally available information, which will be used to calculate the epidemiological costs *C* ^*I*^ and *C* ^*V*^.

#### The parameters (Table 1)

*The rate of infection transmission* **:** *β* is expressed in terms of the basic reproductive ratio of the pathogen *R*_0_ [31]. *R*_0_ corresponds to the average number of secondary infections produced by an infected individual in an otherwise susceptible population and can be understood as the fitness of the pathogen: for a pathogen to invade the population, *R*_0_ must be >1. In our model *R*_0_ has been calculated as:

$$R_{0}=\frac{\left(S_{n}^{\text{*}}+S_{p}^{\text{*}}\right)\beta }{d+\gamma },$$

where $S_{n}^{*}$ and $S_{p}^{*}$ are determined numerically using the system of ordinary differential equations (see section 2.iii) assuming no infection.

**Table 1:** Parameters used and their default values.

Symbols	Meaning	Default values
***b***	Reproductive rate	1 UT^-1^
***d***	Mortality rate	1 UT^-1^
*R*_0_	Basic reproductive ratio	variable
***γ***	Recovery rate	0.1 UT^-1^
***δ*** ^***V***^	Rate—negative side-effects from vaccine	variable
***δ*** ^***I***^	Rate—negative side-effects from infection	1 UT^-1^
***f***	Forgetting rate	5 UT^-1^
***D***	Conformity coefficient	0.7
***ω***_***p*→*n***_	Maximum rate of opinion change (from positive to negative opinion)	2 UT^-1^
***ω***_***n*→*p***_	Maximum rate of opinion change (from negative to positive opinion)	2 UT^-1^
***v***_***n***_	Maximum rate to get vaccinated (negative opinion)	2 UT^-1^
***v***_***p***_	Maximum rate to get vaccinated (positive opinion)	2 UT^-1^
***μ***_***n***_	Weight given to the infection cost (negative opinion)	0.5
***μ***_***p***_	Weight given to the infection cost (positive opinion)	1
$\mu_{n}^{\prime }$	Weight given to the vaccination cost (negative opinion)	1
$\mu_{p}^{\prime }$	Weight given to the vaccination cost (positive opinion)	0.5

UT denotes the unit of time which can be expressed in year or ten years.

Indeed, S* is the number of susceptible individuals, i.e. the number of individuals that are not vaccinated before the emergence of the infection. In our model, people can have two different opinions regarding vaccination which implies two different probabilities to accept the vaccine. Thus the number of susceptible individuals S* depends on the number of individuals who either have a positive or a negative opinion before the emergence of the infectious disease, i.e. $S^{*}=S_{n}^{*}+S_{p}^{*}$ where $S_{n}^{*}$ and $S_{p}^{*}$ represent the population equilibrium of the number of susceptible individuals in the absence of disease.

*The information relating to the costs of infection and vaccination*: This information, available to everyone, is assumed to be summarized by a function of the number of the past and present individuals who have suffered negative side effects from infection and vaccination. This information is used by all individuals in order to measure the relating costs of infection and vaccination, respectively denoted *C* ^*I*^ and *C* ^*V*^. We supposed that this information is generated and memorized by all individuals at a rate *δ* ^*I*^ and *δ* ^*V*^. We also assumed that the information is forgotten at a rate *f*.

At any given point in time, the number of infected individuals is determined by the history of vaccine uptake in the population. Thus, the costs of infection and vaccination depend on both the history of vaccine uptake and the number of infected individuals.

*The rate of vaccination*: Only susceptible individuals can take up vaccination and the decision to vaccinate depends on both an individual opinion and the costs of infection and vaccination. Individuals with a positive opinion vaccinate at the rate *θ*_*p*_; individuals with a negative opinion vaccinate at the rate *θ*_*n*_.

$$\theta_{p}=\upsilon_{p}\;\left(\frac{exp^{x_{p}}}{1+exp^{x_{p}}}\right)\;\text{with}\;x_{p}=\mu_{p}C^{I}-\mu_{p}^{\prime }C^{V}.$$

$$\theta_{n}=\upsilon_{n}\;\left(\frac{exp^{x_{n}}}{1+exp^{x_{n}}}\right)\;\text{with}\;x_{n}=\mu_{n}C^{I}-\mu_{n}^{\prime }C^{V}.$$

*υ*_*p*_ and *υ*_*n*_ represent the maximum rate at which individuals can be vaccinated. While individuals have access to the same epidemiological information *C* ^*I*^ and *C* ^*V*^, the perception of those costs and the subsequent vaccination behaviour is modified by an individual opinion because of a confirmation bias. Confirmation bias is introduced with *μ*, the weight given to the cost of infection and *μ*′, the weight given to the cost of vaccination. During the vaccination decision-making process, individuals with a positive opinion of vaccines give more weight to the infection cost ($\mu_{p}\;>\;\mu_{p}^{\prime }$) while individuals with a negative opinion give more weight to the vaccination cost ($\mu_{n}^{\prime }\;>\;\mu_{n}$).

*The rate of opinion change*: A pro-vaccine individual will become a sceptic at the rate Ω and a sceptic will become a pro-vaccine individual at the rate Ω′.

$$\Omega =\omega_{p\to n}{\;\gamma }_{p\to n}\;c_{n}\;\text{with}\;\gamma_{p\to n}=\left(\frac{exp^{y}}{1+exp^{y}}\right)\;\text{and}\;y=-C^{I}+C^{V}.$$

$$\Omega^{\prime }=\omega_{n\to p}{\;\gamma }_{n\to p}\;c_{p}\;\text{with}\;\gamma_{n\to p}=\left(\frac{{exp}^{y^{\prime }}}{1+{exp}^{y^{\prime }}}\right)\;\text{and}\;y^{\prime }=C^{I}-C^{V}.$$

*ω*_*p*→*n*_ and *ω*_*n*→*p*_ indicate the maximum rates at which an individual changes opinion; Υ_*p*→*n*_ and Υ_*n*→*p*_ indicate resistance to opinion change, which is a function of the costs of infection and vaccination (*C* ^*I*^ and *C* ^*V*^); *c*_*p*_ and *c*_*n*_ indicate the conformity functions, which are non linear frequency-dependent functions, as modelled by [32].

$$c_{p}=a_{p}\;\left[1+D(2a_{p}-1)(1-a_{p})\right]\;\text{where}\;a_{p}=\frac{F}{N},\;\text{with}\;F=S_{p}+I_{p}+R_{p}^{V}+R_{p}^{g}.$$

$$c_{n}=a_{n}\;\left[1+D(2a_{n}-1)(1-a_{n})\right]\;\text{where}\;a_{n}=\frac{N_{g}}{N},\;\text{with}\;N_{g}=S_{n}+I_{n}+R_{n}^{V}+R_{n}^{g}.$$

*F* and *N*_*g*_ indicate the number of pro-vaccines and sceptical individuals in the entire population denoted *N*; *D* is the conformity coefficient. The more common is a given opinion, the more often it is adopted.

*The parameter time unit*: It is calibrated in relation to the birth and death rates. We generally set the birth and death rates at the value 1 which, in the case of human populations, could correspond to a time unit of the order of a year or ten years.

#### The dynamic

The dynamic of the system can be described by the following set of ordinary differential equations:

$$\frac{dS_{p}}{dt}=bF+\Omega^{\prime }S_{n}-\Omega S_{p}-\theta S_{p}-\beta S_{p}\left(I_{p}+I_{n}\right)-dS_{p},$$

$$\frac{dS_{n}}{dt}=bN_{g}-\Omega^{\prime }S_{n}+\;\Omega S_{p}-\theta^{\prime }S_{n}-\beta S_{n}\left(I_{p}+I_{n}\right)-dS_{n},$$

$$\frac{dR_{p}^{V}}{dt}=\theta S_{p}\;+\Omega^{\prime }R_{n}^{V}-\Omega R_{p}^{V}-dR_{p}^{V},$$

$$\frac{dR_{n}^{V}}{dt}=\theta^{\prime }S_{n}\;+\Omega R_{p}^{V}-\Omega^{\prime }R_{n}^{V}-dR_{n}^{V},$$

$$\frac{dI_{p}}{dt}=\beta S_{p}\left(I_{p}+I_{n}\right)+\Omega^{\prime }I_{n}-\Omega I_{p}-\gamma I_{p}-dI_{p},$$

$$\frac{dI_{n}}{dt}=\beta S_{n}\left(I_{p}+I_{n}\right)+\Omega I_{p}-\Omega^{\prime }I_{n}-\gamma I_{n}-dI_{n},$$

$$\frac{dR_{p}^{g}}{dt}=\gamma I_{p}+\Omega^{\prime }R_{n}^{g}-\Omega R_{p}^{g}-dR_{p}^{g},$$

$$\frac{dR_{n}^{g}}{dt}=\gamma I_{n}+\Omega R_{p}^{g}-\Omega^{\prime }R_{n}^{g}-dR_{n}^{g},$$

$$\frac{dC^{V}}{dt}=\delta^{V}\left(R_{n}^{V}+R_{p}^{V}\right)\;-fC^{V},$$

$$\frac{dC^{I}}{dt}=\delta^{I}\left(I_{p}+I_{n}\right)\;-fC^{I}.$$

### Nested models

The full model described above combines five elements: (1) the characterization of individuals by their epidemiological status (SIR model); (2) the characterization of individuals by their opinion; (3) a globally available information on the infection and vaccination costs; (4) the interpretation of epidemiological costs as a function of opinion through confirmation bias and (5) the transmission of opinion through conformism. To evaluate the significance of each element in shaping the dynamics of infection and vaccination coverage, we broke down the full model into several nested models differing by 1 element at a time. Specifically, we investigate the same range of parameter values (Table 1) for the five nested models in order to be able to detect the effect of each element (Table 2):

**Model 1:** a classic SIR model without the belief component.

**Model 2:** a model that characterizes individuals by both their epidemiological status and their opinion. Vaccination rate is fixed (*θ*_*p*_ = *υ*_*p*_; *θ*_*n*_ = *υ*_*n*_) and individuals with a positive opinion vaccinate more quickly than those with a negative opinion (*υ*_*p*_ > *v*_*n*_). By doing so, vaccination behaviour is a function of opinion but not confirmation bias (*μ*_*p*_ = *μ*_*n*_ = 1), i.e. the difference in vaccination rates between individuals with opposed opinions is not a function of the number of infected individuals and vaccination coverage. The rate of opinion change is also fixed (Ω = *ω*_*p*→*n*_ = Ω′ = *ω*_*n*→*p*_).

**Model 3:** This model includes the globally available information on the number of individuals suffering negative side effects from the infection *C* ^*I*^ and vaccination *C* ^*V*^. This enables to calculate the epidemiological costs.

**Model 4:** This model includes confirmation bias, i.e. the biased perception of costs by individual opinion. In Models 1–3 confirmation bias is not considered so *μ* = *μ*′ regardless of the opinion of individuals.

**Model 5**: This model includes conformism and corresponds to the full model. In Models 1–4, conformism is not considered so *c*_*p*_ = *c*_*n*_ = 1.

**Table 2:** Components of the different nested models.

Models	SIR model	Opinions	Disease and vaccine costs	Confirmation bias	Conformism
Model 1	X				
Model 2	X	X			
Model 3	X	X	X		
Model 4	X	X	X	X	
Model 5	X	X	X	X	X

#### Numerical simulation

We performed all simulations using Mathematica 9.0 [33]. First, 500 susceptible positive individuals and 500 susceptible negative individuals are introduced. We ran 100 000 iterations with a time step of 10^−4^, the number of iterations needed to reach a stable equilibrium, to determine the number of susceptible individuals ($S_{n}^{*}$ and $S_{p}^{*}$) and calculate the rate of infection transmission *β* with *R*_0_ in the absence of disease. Second, after *β* was obtained, we introduced two infected individuals, one of each opinion (*N* = 1002 individuals). We ran 1,000 000 iterations with a time step of 10^−4^ which corresponds to 100 time units (e.g. 100 years), to ensure that we capture the complete dynamic. To evaluate the role of each component of the full model in shaping the dynamics of infection and vaccination coverage, we analysed and compared the results of the nested models. We run all models for values of *R*_0_ ranging from 0 to 10 in steps of 0.5. All other parameter values are detailed in Table 1.

## Results

**Fig 2:**
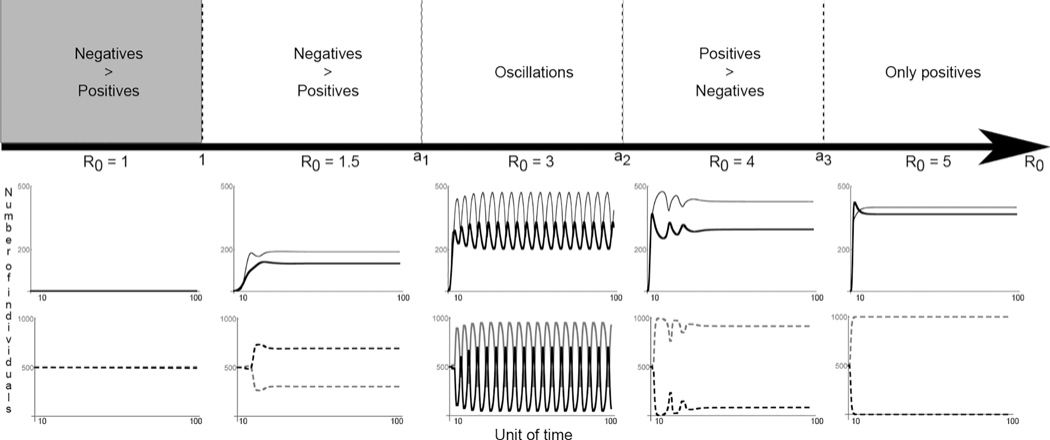
Infection and opinion dynamics as a function of the reproductive rate of the infection (model 5). For each value of R_0_ (number of secondary infections which can be understood as the fitness of the pathogen; it must be >1 for the pathogen to invade the population), the time-series of the dynamics of the infection (top panel; the thin line corresponds to the number of infected individuals and the thick line corresponds to the number of vaccinated individuals) and opinion (bottom panel; the dashed grey line indicates the number of individuals who have a positive opinion and the dashed black line indicates individuals who have a negative opinion) are depicted for a rate of negative side effects from vaccination *δ* ^*v*^ = 0.7. Alternative changes of opinions of vaccination and oscillations in vaccination coverage emerge for intermediate values of R_0_. When the reproductive rate of the disease is large (R_0_ >4), the negative opinion of vaccination disappears and vaccination coverage, while reaching high levels, does not reach herd immunity. This is because the infection spreads too quickly. More generally, four dynamics can be observed and a_1_ to a_3_ represent their limits which can be moved depending of the values of *δ* ^*v*^ considered.

**Fig 3:**
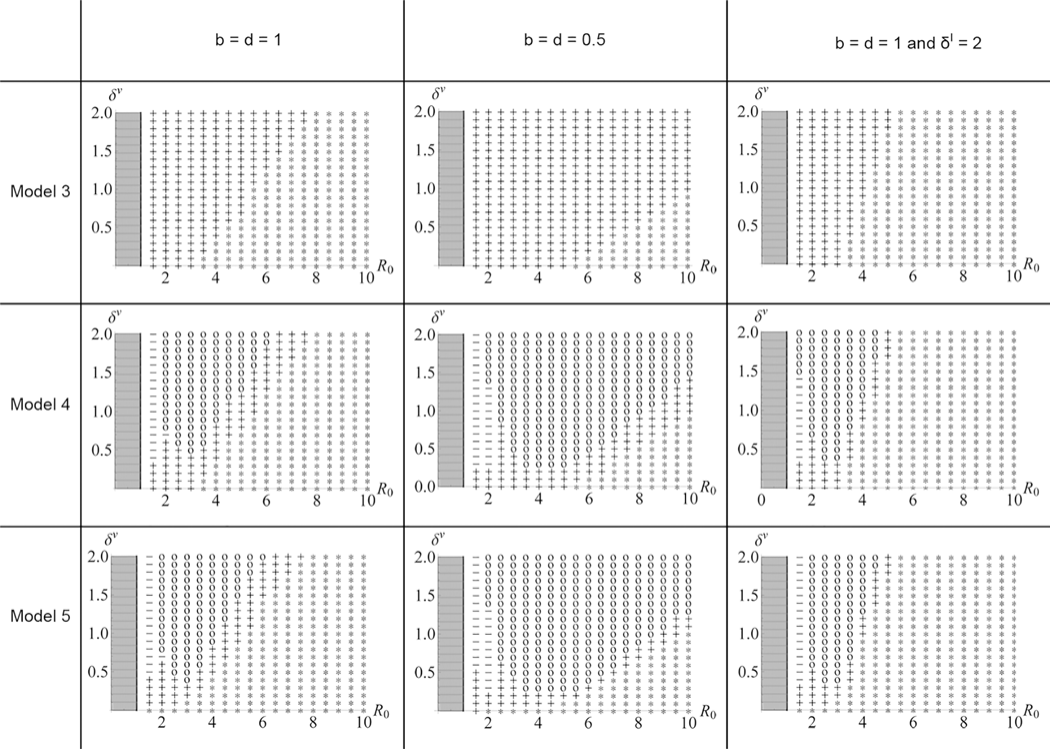
Confirmation bias, conformism and the emergence of oscillations in vaccination coverage. The emergence of oscillations (o) is depicted as a function of an infection’s reproductive rate (R_0_) and the rate of negative side effects from vaccination (*δ* ^*v*^). The emergence of oscillations is contingent on the inclusion of confirmation bias but not conformism and is observed for intermediate values of R_0_. Three models are considered: Model 3 (without either confirmation bias or conformism); Model 4 (including confirmation bias) and Model 5 (including both confirmation bias and conformism). If no oscillations emerge, three dynamics can be noted for the distribution of opinions of vaccination: (i) coexistence of 2 opinions with a greater number of individuals with a positive opinion (+), (ii) coexistence of 2 opinions with a greater number of individuals with a negative opinion (-), (iii) all individuals have a positive opinion (*). The models are analysed for 2 different generation times (birth (*b*) = death (*d*) = 1 and 0.5) and for different values of the rate of negative side effects from infection (*δ* ^*I*^). 1 million iterations were run with a time step of 0.0001 which correspond to 100 unit of time.

**Fig 4:**
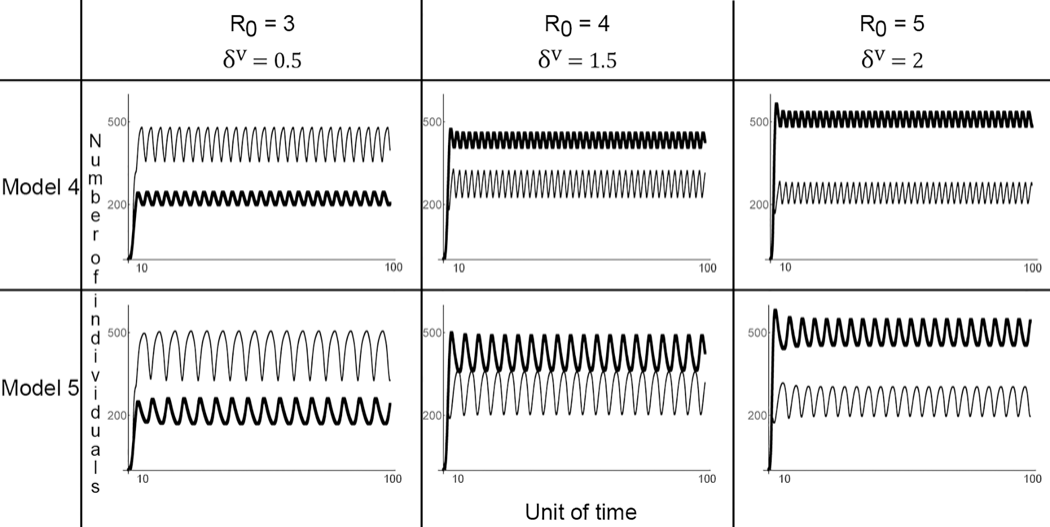
Conformism and oscillations in vaccination coverage. The evolution of the number of infected (thin line) and (thick line) vaccinated individuals is depicted. Conformism (illustrated by Model 5) increases the amplitude of oscillations and slows down the rate at which alternative opinions of vaccination alternate. The situation is indicated for infections that are moderately infectious (with a reproductive ratio (R_0_) varying from 3 to 5) and with the rate of negative side effects from the vaccination (*δ* ^*V*^) varying from 0.5 to 2.

Our results are threefold: (i) for all parameter values investigated, the infection is never eradicated under voluntary vaccination, i.e. vaccination coverage never reaches herd immunity, that is, the threshold of vaccination coverage needed for stopping the spread of the infection; (ii) the dynamics of infection and vaccination coverage depend on the presence/absence of a polymorphism for the opinion of individuals, which is a function of the transmissibility potential of the pathogen (*R*_0_) (Fig 2). If the infection is not contagious (*R*_0_ < 1), all individuals become sceptical and vaccination coverage remains low; if infection is highly contagious (*R*_0_ >> 1), all individuals become pro-vaccines but herd immunity is never reached because the infection spreads too quickly. Yet, vaccination coverage remains high. For intermediate values of *R*_0_, both sceptical and pro-vaccine individuals coexist and oscillations in vaccination coverage are observed (Fig 3); (iii) the rule “imitate the majority” is not necessary for oscillations to appear. Rather, the transmission of opinions through conformism increases the delay between changes in epidemiological parameters and the behavioural responses to those changes (Fig 4).

### What is the role of R_0_ for the dynamics of vaccination coverage?

Various dynamics are observed depending on the reproductive rate of the infection because the value of *R*_0_ influences the distribution of opinions in the population. For infections that spread very quickly in the population (*R*_0_ >> 1), only “pro-vaccines” individuals persist. For intermediate values of *R*_0_, a stable coexistence of “sceptics” and “pro-vaccine” individuals is observed with three possible cases: (1) at equilibrium, a negative opinion of vaccination is more frequent; (2) at equilibrium, a positive opinion of vaccination is more frequent; (3) which opinion is the most frequent oscillates over time (Fig 2). Oscillations are only observed when the cost of vaccination (*C* ^*V*^) is high enough, i.e. when a sufficient number of individuals are vaccinated and suffer from side effects. The influence of other parameters on the emergence of oscillations is depicted Fig 3.

### The emergence of oscillations in vaccination coverage

#### The role of cognition (confirmation bias and conformism)

We compared nested models to disentangle the role of confirmation bias and conformism on the distribution of opinions towards vaccination and subsequently, the emergence of oscillations in vaccination coverage. We found that oscillations can only emerge when both negative and positive opinions of vaccination coexist in the population and when a confirmation bias is included (Models 4 & 5, see also S1 Fig for various values of *μ*_*p*_, *μ*_*n*_ and *μ*_*p*_/*μ*_*n*_). To confirm the significance of confirmation bias, we reran the full model while excluding confirmation bias (*μ*_*p*_ = *μ*_*n*_): oscillations do not appear (S2 Fig). Then we found that conformism does not affect the emergence of oscillations in the dynamics of infection and vaccination coverage (Fig 3). Rather, oscillations become less frequent and more ample because conformism increases the time necessary for the least frequent opinion to invade the population (Fig 4). To confirm that conformism does not produce oscillations, we compared the results obtained for the full model with and without biased social transmission (*D* = 0.7 and *D* = 0, respectively): when confirmation bias is considered oscillations do appear in both situations (S2 Fig).

#### The role of epidemiology (vaccination and infection costs)

Once opinion heterogeneity and confirmation bias are considered, the emergence of oscillations in vaccination coverage depends on the ratio between the costs of vaccination and infection. For instance, in the case of an infection with a low epidemic potential (*R*_0_ is low) and few individuals infected, both the infection cost and the vaccination cost are low because only a few individuals suffer negative side effects. Consequently, vaccination coverage remains low and stable over time. For infections with a high reproductive rate (*R*_0_ is high), the infection invades the population more rapidly than vaccination behaviour. Thus, the cost of infection is always higher than the cost of vaccination. Similarly, when the cost of vaccination is low, oscillations do not emerge because the cost of vaccination is always lower than that of infection.

## Discussion

Despite evidence that vaccines are safe, vaccine scares are repeatedly observed in various populations and cause the resurgence of diseases otherwise near eradication (e.g. measles). A key factor in driving vaccination decisions is how epidemiological parameters, in particular the costs associated with infection and vaccination, are perceived. Although perception may be based on epidemiological information, the success of anti-vaccination movements also suggests that individuals vary in the way they interpret scientific evidence. People differ in their opinion of vaccination because of their past experience with immunization, their trust in the state or in the medical profession, the opinion of their parents or the most common opinion in their cultural environment, irrespective of a cost-benefit analysis based on the epidemiology [34]. In this paper, we deviate from previous theoretical studies positing that individuals are economic agents maximizing their health [4,5,35–39] to consider that individuals make decisions as result of cognitive biases. Specifically, we developed a behaviour-incidence model in which individuals can be pro-vaccines or sceptical, their opinion influencing how the costs of infection and vaccination are perceived. Overall, the model predicts the commonly observed dynamics of vaccination coverage, i.e. the failure to reach herd immunity as well as oscillations in vaccination coverage (periods of increase in vaccination coverage followed by disease outbreaks). The results have implications for considering how the interaction between human cognition and the epidemiology may lead to difficulties in reaching and/or maintaining population level immunity.

### The emergence of oscillations

D’Onofrio et al. [40] showed that assuming a memory of the information related to the costs of infection and vaccination can generate oscillations in a SIR model without cognitive bias. Comparing the behaviour of our model with or without cognitive bias we have shown that the confirmation bias is necessary for the emergence of oscillations in the dynamics of infection and vaccination coverage, at least in the parameter range we explore. Our model and that of d’Onofrio *et al*. differ by several hypotheses, which could explain why the conditions necessary for the emergence of oscillations differ between the two models. D’onofrio et al. assumed that memory is fading following an exponential rate and that the infection rate is frequency dependent while we supposed a memory fading at a linear rate and a density dependent infection rate. In all cases, our model is the first to show that confirmation bias can strongly facilitate cyclical dynamics.

The propensity to seek out information that confirms one’s pre-existing belief, and the possibility for individuals to change their opinion as a function of the number of vaccinated individuals (i.e. confirmation bias) can thus explain variation into vaccination coverage. When vaccination coverage increases, the cost of vaccination increases too as more individuals suffer vaccination side effects. It leads to a rise in the number of individuals who become sceptical about vaccines. Those “sceptics” are more likely to overestimate the cost of vaccination as compared to that of infection, and thus when most of the population is vaccinated; sceptics perceive the infection as harmless. Eventually, this phenomenon leads to a decrease in vaccination coverage and subsequently, to the resurgence of diseases. In turn, as the number of infected individuals increase, a “pro-vaccine” opinion spreads leading to an increase in vaccination coverage. Those results are not different from those obtained in other models where vaccine cost appears large in comparison to that of infection as the population approaches herd immunity [41]. However, in some previous theoretical studies, the cost of vaccination does not change as a function of the number of people vaccinated. Rather, the increase in vaccination cost is imposed using an exogenous description of the evolution of vaccine risk [41], the inclusion of a cyclical period to simulate a vaccine scare [27,39] or through a decrease in the accessibility of vaccines [37,42,43]. While previous studies showed that a vaccine scare could lead to a decrease in vaccination coverage, the present model explicitly articulates a mechanism creating a vaccine scare (i.e. when the cost of vaccination is perceived to be greater than that of the infection cost). The results reported here are contingent on the assumption that the vaccination decision-making process is sensitive to a confirmation bias. This cognitive phenomenon is only one among several, however, as other heuristics have been documented in previous research on vaccine acceptance [25,44], for instance omission bias (when people prefer act of omission over act of commission, even if the outcome of omission is worse) or ambiguity aversion (aversion of ambiguous outcomes). Further work explicitly comparing the significance of various cognitive biases may yield additional insight into the role of cognition in generating vaccine scares.

### Social learning and vaccinating decision-making

A number of previous studies have investigated the role of social interactions in shaping the emergence of oscillations in vaccination coverage [13,17,27,29,35,45,46]. This is because vaccination coverage can be relatively stable in the absence of a vaccine scare and the feedback between the number of infected individuals and vaccination behaviour appears insufficient in accounting for the delay between reaching high vaccination coverage and the emergence of a scare. To account for the role of social interactions, behavioural epidemiology models usually consider that individuals imitate the most successful strategy among their neighbouring contacts [39,47] and such models are better than models considering a homogenous population for fitting data on vaccine scares [41]. The way social learning is modelled explicitly considers that individuals are rational agents maximizing their payoff. Individuals can be influenced by social interactions in different ways, however, and the role of social influence need not be connected to epidemiological risks [48]. For instance, it has been argued that mothers taking their children for vaccination has become part of their “habitus” (*sensu* Bourdieu, in [34]). Mothers elicit to vaccinate their children because “*everyone else does*” and ethnographic studies on the acceptance of vaccines in various populations revealed that peer opinion matters [34]. In the model developed here, individuals adopt the most common opinion in the population through the phenomenon of conformism. Conformism does not generate oscillations in vaccination coverage but increases the amount of time needed for a change in epidemiology to generate a cultural response. The role of conformism in increasing the time for cultural change has been observed in previous studies [39].

### Limitations

First, the model presented here assumes that both infection and health behaviour are transmitted within a homogeneously mixing population, and those are simplifying assumptions. Humans are connected with a limited number of contact [49] and a number of studies has considered that social heterogeneity influences disease transmission [14,50,51], risk perception [15,20,52] and vaccination behaviour [15,48,53]. Clustered occurrence of beliefs can lead to the clustered occurrence of disease [54]. Nevertheless, in the context of information transmission, the assumption according to which the population is homogeneously mixed is valid if we focus on the role of the media, i.e. information that is globally available to everyone. The media has had a central role in disseminating vaccine scares and shaping the perception of disease severity [18]. The media also enables anti-vaccination movements to have a strong impact on vaccination decision-making [7,55]. Yet, if the proposed model may be appropriate for exploring the role of conformism, it is insufficient to explore other social transmission processes such as those resulting from the role of opinion leaders (e.g. cultural transmission through prestige-bias [56]). Religious leaders have been shown to be highly influential in promoting vaccine refusal in their communities (reviewed in [34]) and the role of prestige-biased cultural transmission for either accelerating the pace of change or stopping it altogether remains to be investigated. Second, one may argue that since the costs depend on the number rather than the frequency of individuals suffering side effects, the model is more likely to inform on information that is gathered during social interactions than knowledge acquired via the media. The media report both percentages and anecdotic evidence of side effects associated with vaccination and both are likely to be determinant in driving the formation of opinion. Yet, the relative role of each type of information deserves further investigation. Finally, we assumed that the rate of opinion change, while a function of epidemiological costs, is independent of the SIR class of the individual. However, an individual suffering infection may be less likely to switch from a positive to a negative opinion and thus we may have overestimated the occurrence of oscillations. Note however that for diseases with a strong epidemic potential, the cost of infection is always higher than that of vaccination and therefore oscillations do not emerge.

This study, along a few others [22,28,29,48], offers an alternative to payoff maximization for understanding the emergence of oscillations in vaccination coverage. It shows that a decrease in vaccine uptake when a disease is near eradication may not only emerge because of individual free riding but because of cognitive biases that maintain heterogeneity in how people perceive risks. While this study presents limitations that need to be explored in future work, i.e. considering heterogeneity in contact patterns as well as additional cognitive biases, we show that alternative notions of rationality, in particular that of an ecological rationality whereby individuals use simple cognitive heuristics, offer interesting new avenues for modelling vaccination behaviour.

## Supporting Information

S1 Fig The effect of confirmation bias on oscillations. The effect of confirmation bias (i.e. with *μ* the weight given to the cost of infection and *μ*′ the weight given to the cost of vaccination) on the dynamics of susceptible (solid grey line), infected (thin line) and vaccinated individuals (thick line) is depicted. The amplitude and the frequency of oscillations are modified when the difference between *μ* and *μ*′ is high. Indeed, the bigger the difference between *μ* and *μ*′, the more the amplitude of oscillations increases and the more the frequency decreases. The situation is indicated for infections that are moderately infectious, with 2 reproductive ratio R_0_, 3 and 6 and with a rate of negative side effects from the vaccination *δ* ^*V*^ = 1.
(TIF)

S2 Fig The effect of conformism and confirmation bias on the appearance of oscillations. The effect of conformism biased (*D* = 0.7) and unbiased (*D* = 0) is depicted with and without confirmation bias. The number of susceptible individuals is represented by the solid grey line, the infected individuals by the thin line and the number of vaccinated individuals by the thick line. Without confirmation bias (*μ* = *μ*′ = 1), oscillations do not appear for both types of conformism (biased and unbiased). When the confirmation bias is added, oscillations do appear with no significant differences between the biased and unbiased conformism. The situation is indicated with the reproductive ratio R_0_ = 3 and with a rate of negative side effects from the vaccination *δ* ^*V*^ = 1.
(TIF)
