# Correction: Beyond Rational Decision-Making: Modelling the Influence of Cognitive Biases on the Dynamics of Vaccination Coverage

**DOI:** 10.1371/journal.pone.0167842

**Published:** 2016-12-01

**Authors:** Marina Voinson, Sylvain Billiard, Alexandra Alvergne

There is an error in the sixth sentence of the Abstract. The correct sentence is: As a result of confirmation bias, sceptical individuals (negative opinion) overestimate vaccination cost while pro-vaccines individuals (positive opinion) overestimate infection cost.
