# Supplementary figures and images for: Beyond Rational Decision-Making: Modelling the Influence of Cognitive Biases on the Dynamics of Vaccination Coverage
Source: PLoS One. 2015 Nov 23;10(11):e0142990. doi: 10.1371/journal.pone.0142990 (PMC4657916; doi:10.1371/journal.pone.0142990)

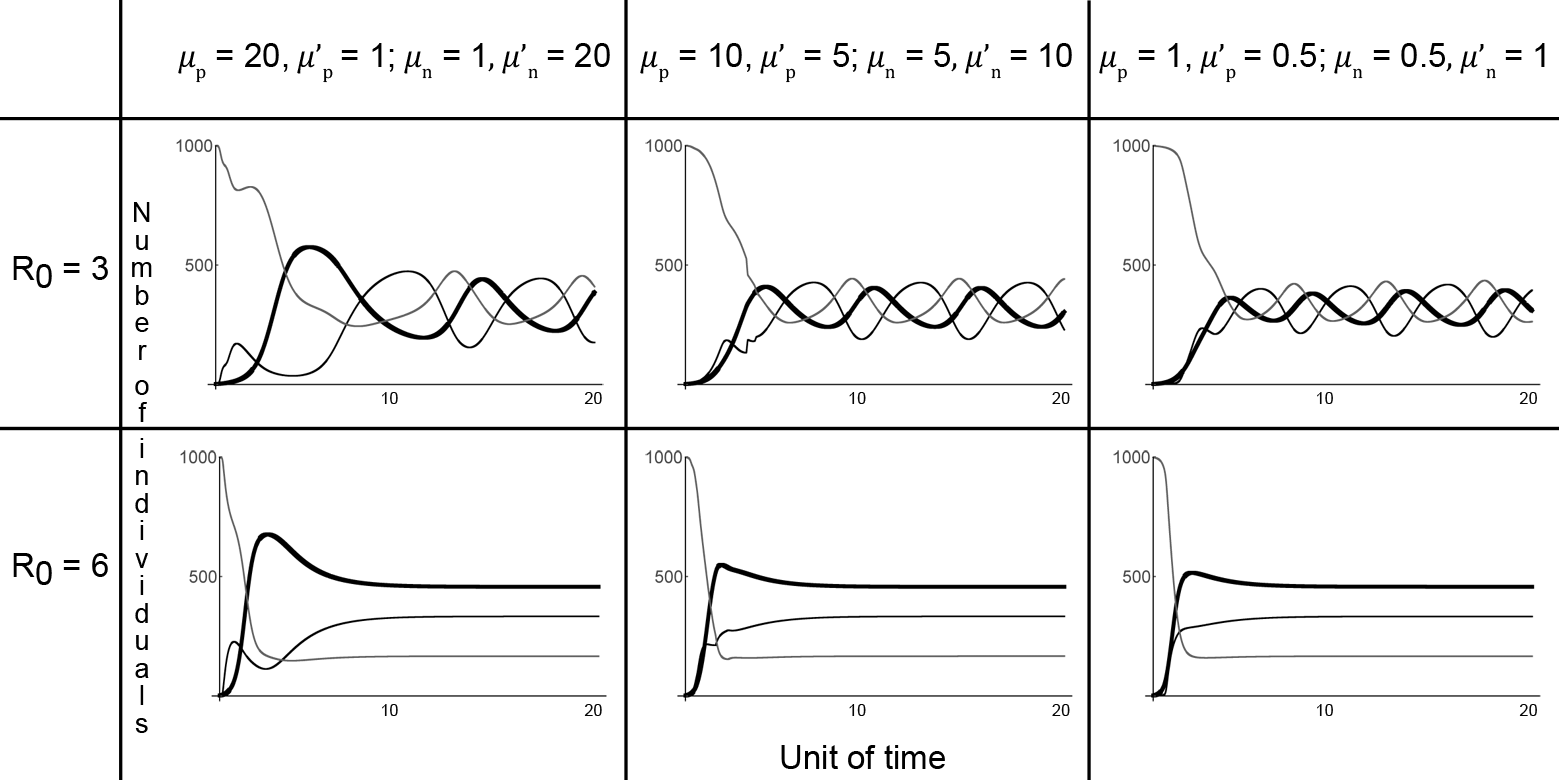

Supplement: S1 Fig — The effect of confirmation bias (i.e. with μ the weight given to the cost of infection and μ′ the weight given to the cost of vaccination) on the dynamics of susceptible (solid grey line), infected (thin line) and vaccinated individuals (thick line) is depicted. The amplitude and the frequency of oscillations are modified when the difference between μ and μ′ is high. Indeed, the bigger the difference between μ and μ′, the more the amplitude of oscillations increases and the more the frequency decreases. The situation is indicated for infections that are moderately infectious, with 2 reproductive ratio R0, 3 and 6 and with a rate of negative side effects from the vaccination δ V = 1. (TIF) [file pone.0142990.s001.tif]

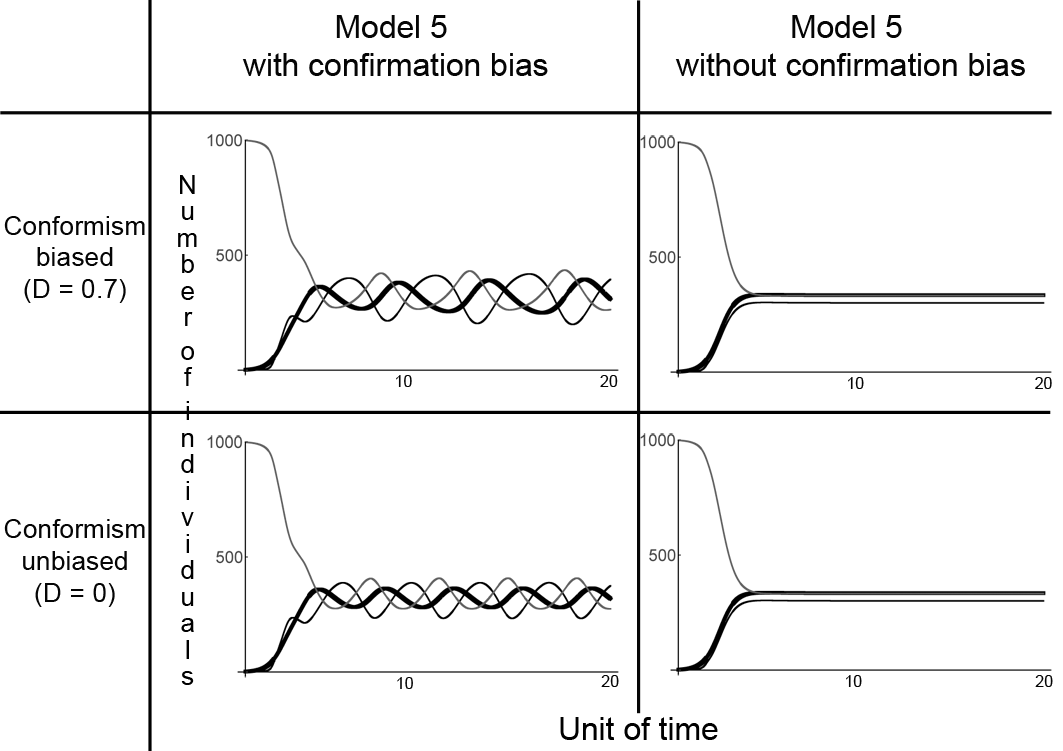

Supplement: S2 Fig — The effect of conformism biased (D = 0.7) and unbiased (D = 0) is depicted with and without confirmation bias. The number of susceptible individuals is represented by the solid grey line, the infected individuals by the thin line and the number of vaccinated individuals by the thick line. Without confirmation bias (μ = μ′ = 1), oscillations do not appear for both types of conformism (biased and unbiased). When the confirmation bias is added, oscillations do appear with no significant differences between the biased and unbiased conformism. The situation is indicated with the reproductive ratio R0 = 3 and with a rate of negative side effects from the vaccination δ V = 1. (TIF) [file pone.0142990.s002.tif]
